# Comparative Study of Male and Female Patients Undergoing Frozen Elephant Trunk Total Arch Replacement

**DOI:** 10.3390/jcm12196327

**Published:** 2023-10-01

**Authors:** Julia Benk, Tim Berger, Stoyan Kondov, Matthias D’Inka, Magdalena Bork, Tim Walter, Philipp Discher, Bartosz Rylski, Martin Czerny, Maximilian Kreibich

**Affiliations:** Department of Cardiovascular Surgery, Heart Centre Freiburg University, Faculty of Medicine, University of Freiburg, 79106 Freiburg, Germany; tim.berger@uniklinik-freiburg.de (T.B.); stoyan.kondov@uniklinik-freiburg.de (S.K.); matthias.dinka@uniklinik-freiburg.de (M.D.); magdalena.bork@uniklinik-freiburg.de (M.B.); tim.walter@uniklinik-freiburg.de (T.W.); philipp.discher@uniklinik-freiburg.de (P.D.); bartosz.rylski@uniklinik-freiburg.de (B.R.); martin.czerny@uniklinik-freiburg.de (M.C.); maximilian.kreibich@uniklinik-freiburg.de (M.K.)

**Keywords:** frozen elephant trunk (FET), sex difference, aortic dissection, aortic aneurysm, arch surgery

## Abstract

Background: Our aim was to investigate outcomes and long-term survival in male and female patients after frozen elephant trunk (FET) total arch replacement. Methods: Between March 2013 and January 2023, 362 patients underwent aortic arch replacement via the FET technique. We compared patient characteristics and intra- and postoperative data between male and female patients. Results: Male patients were significantly younger (*p* = 0.012) but revealed a higher incidence of coronary artery disease (*p* = 0.008) and preoperative dialysis (*p* = 0.017). More male patients presented with type A aortic dissections (*p* = 0.042) while more female patients had aortic aneurysms (*p* = 0.025). The aortic root was replaced in significantly more male patients (*p* = 0.013), resulting in significantly longer cardiopulmonary bypass duration (*p* < 0.001) and operative times (*p* < 0.001). There were no statistically significant differences in postoperative outcome parameters including in-hospital mortality (*p* = 0.346). However, new in-stent thrombus formation was significantly more frequent in female patients (*p* = 0.002). Age in years (odds ratio (OR): 1.026, *p* = 0.049), an acute pathology (OR: 1.941, *p* = 0.031) and preoperative dialyses (OR: 3.499, *p* = 0.010) were predictive for long-term mortality in our Cox regression model, sex (*p* = 0.466) was not. There was no statistical difference in overall survival (log rank: *p* = 0.425). Conclusions: Female patients are older but reveal fewer cardiovascular risk factors; aneurysms are more common in female than male patients. As female patients undergo concomitant surgical procedures less often, their operative times are shorter. While survival and outcomes were similar, female patients suffered from postoperative new in-stent thrombus formation significantly more often.

## 1. Introduction

The frozen elephant trunk (FET) procedure has become an effective and widespread treatment for patients with thoracic aortic pathologies involving the aortic arch, with good postoperative outcomes in patients with acute or chronic aortic dissections, aortic aneurysms or penetrating aortic ulcers. This hybrid procedure combines an open surgical total aortic arch replacement with distal endovascular stent-graft implantation in the descending aorta. While the procedure remains major surgery, good in-hospital results have been reported. However, during follow-up, a substantial risk for secondary planned and unplanned aortic reinterventions has been identified. Hence, close and continuous follow-up of these patients following the FET procedure has been recommended [1,2].

Female patients have a higher risk profile and worse outcome after cardiac, non-aortic surgery than male patients. Several studies investigating coronary artery bypass graft and valve surgery showed worse outcomes for female patients in short- and long-term survival. Several influencing factors, like the relative physical frailty of women or advanced stage of disease at operation due to later diagnosis, have been discussed [3,4]. However, the reasons for this difference between male and female patients still remain relatively unclear. Our aim was therefore to investigate the outcomes and long-term survival in male and female patients following FET total arch replacement.

## 2. Materials and Methods

Ethical statement. Our institutional review committee approved this retrospective study, and the need for informed consent was waived (number: 20-1302; approval date: 4 February 2021).

Patients and follow-up protocol. Between March 2013 and January 2023, 362 patients underwent FET total arch replacement in one aortic centre currently performing over 60 total aortic arch procedures per annum (as of 2022). These patients underwent a median follow-up of 10 (first quartile: 2; third quartile 30) months. All patients were routinely followed up after six months, 12 months and yearly thereafter in our dedicated aortic clinic. Computed tomography angiography (CTA) scans were done preoperatively, before discharge, during every follow-up visit and when clinically warranted.

Surgical approach and technique. Our standardised, integrated surgical management of the FET technique has already been reported [5]. In short, we carry out a full sternotomy and generally cannulate the right axillary artery for arterial inflow during cardiopulmonary bypass. Usually, 400 IU heparin per kilogram bodyweight is given and we aim for an activated clotting time (ACT) over 400 s during cardiopulmonary bypass. Any concomitant procedures (such as valve, aortic root or coronary artery) take place while the patients are cooled down to a target core body temperature of 25 °C. We routinely apply cold-blood cardioplegia or the beating-heart technique (using 300 mL of normothermic myocardial perfusion) [5]. Bilateral cerebral perfusion is normally used, and we carry out trilateral antegrade cerebral perfusion liberally (additional cannulation of the left subclavian artery) when needed. For this reason, our present preoperative work-up includes a CTA of the supra-aortic vessels including the Circle of Willis. Zone 2 is our standard anastomosis site for FET implantation, and we now employ the short version (100 mm) of the Thoraflex (Terumo Aortic, Inchinnan, UK) hybrid-graft exclusively. In case of classical aneurysm formation, we oversize the stent-graft component by 10% at the distal landing zone and, in case of aortic dissections, we avoid oversizing and choose the FET stent-graft size according to institutional standards. We do not routinely implant cerebrospinal fluid drainage before surgery. As post-operative anticoagulation we recommend acetylsalicylic acid 100 mg per day following the FET procedure. In case there is another indication for oral anticoagulation, we believe oral anticoagulation is sufficient and do not recommend addition acetylsalicylic acid.

Indication for additional cardiac procedures. Every additional surgical procedure was carried out according to the 2014 ESC guidelines [6]. In selected cases of young patients and/or those with connective tissue disease, especially if the aortic root wall looked especially thin intraoperatively, root replacement was done in root diameters smaller than the guideline’s threshold in an anticipatory manner.

Data collection and definition of parameters. Data were collected retrospectively relying on our prospectively maintained aortic database. Acute aortic dissection was defined as a symptom onset fewer than 14 days before hospital admission and was classified as chronic if symptoms had occurred >14 days beforehand. We followed the TEM classification to categorise aortic dissections (Type A, Type B, Type non-A non-B) [7]. The modified Rankin Scale (mRS) was used to classify postoperative stroke severity [8]. Consulting neurologists evaluated all strokes. Postoperative strokes causing no clinical symptoms (mRS 0), no significant disability (mRS 1) or slight disability (mRS 2) were classified as non-disabling postoperative strokes.

Statistical Analysis. Data are presented as absolute and relative frequency or as median (first quartile, third quartile). The Student’s *t*-test or Mann–Whitney U-test was used to compare continuous variables as appropriate. Normality was assessed using the Kolmogorov–Smirnov test. Categorical variables were compared using the Chi-squared test. In case of small group sizes (*n* < 5), Fisher’s Exact test was used. We ran Cox regression analyses to assess the influence of clinically selected variables on overall mortality (selected variables: sex, age in years, acute pathology, redo case and preoperative dialysis) and the Kaplan–Meier method was used to analyse and compare overall survival. Statistical analyses were done using IBM SPSS Statistic 21.0 for Windows.

## 3. Results

Patient characteristics. Male patients were significantly younger (female: 68 (0, 77) vs. male: 65 (58, 73), *p* = 0.012), but presented with a higher incidence of coronary artery disease (female: *n =* 28 (22%) vs. male: *n =* 83 (36%), *p* = 0.008) and preoperative dialysis (female: *n =* 8 (6%) vs. male: *n =* 35 (15%), *p* = 0.017). Patient characteristics are summarised in Table 1.

Preoperative characteristics. As Table 2 shows, more male patients underwent type A aortic dissections (female: *n =* 40 (31%) vs. male: *n =* 99 (42%), *p* = 0.042) and more male patients were treated for acute type A (female: *n =* 14 (11%) vs. male: *n =* 50 (21%), *p* = 0.014) or type B (female: *n =* 15 (12%) vs. male: *n =* 10 (4%), *p* = 0.009) dissections than female patients. An aortic aneurysm involving the aortic arch was the indication for FET implantation in significantly more female patients (female: *n =* 50 (39%) vs. male: *n =* 65 (28%), *p* = 0.025).

Surgical details. Intraoperative characteristics are summarised in Table 3. More male patients underwent concomitant aortic root replacement (female: *n =* 17 (13%) vs. male: *n =* 59 (26%), *p* = 0.013) because of a higher incidence of male patients requiring a valved conduit (female: *n =* 9 (7%) vs. male: *n =* 34 (15%), *p* = 0.041). We also noted a tendency towards more coronary artery bypass surgery in male patients, but the difference failed to reach statistical significance (female: *n =* 13 (10%) vs. male: *n =* 41 (18%), *p* = 0.088). The males’ higher incidence of concomitant cardiac surgery resulted in longer cardiopulmonary bypass duration (female: *n =* 189 (162, 232) vs. male: 218 (184, 261), *p* < 0.001) and longer operative times (female: *n =* 361 (300, 417) vs. male: 396 (345, 474), *p* < 0.001). There was no statistically significant difference in perioperative transfusions.

**Figure 1:**
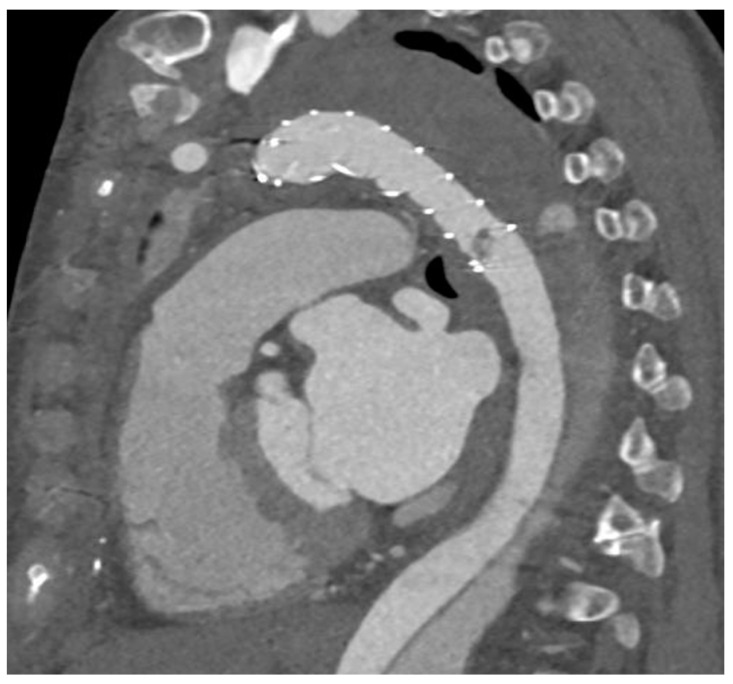
Representative computed-tomographic-angiographic depiction of a newly diagnosed postoperative thrombus formation within the frozen elephant trunk stent-graft.

**Figure 2:**
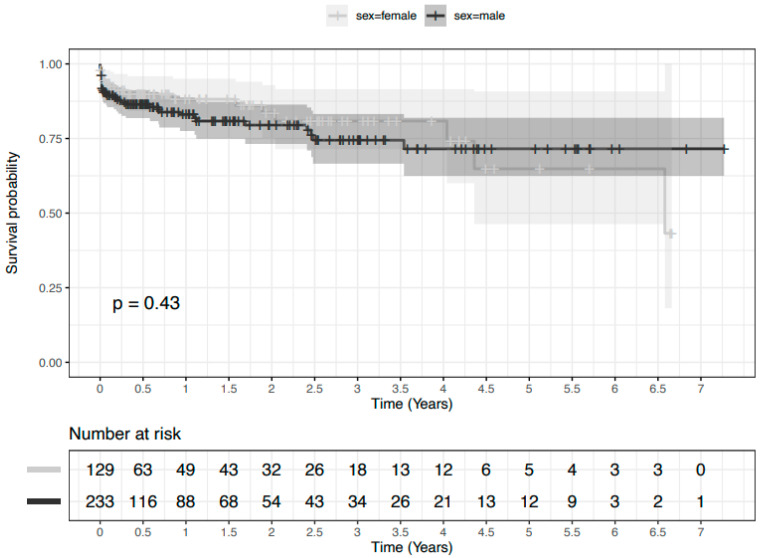
Kaplan–Meier curve of overall survival of male (dark grey) and female (light grey) patients following frozen elephant trunk total arch replacement. The 95% confidence interval is depicted. Log rank: *p* = 0.425. Time in years.

Outcome characteristics. Postoperative outcomes were similar between male and female patients including in-hospital mortality (female: *n =* 9 (7%) vs. male: *n =* 24 (10%), *p* = 0.346) as in Table 4. However, a new postoperative in-stent thrombus formation (Figure 1) was diagnosed in significantly more female patients (female: *n =* 13 (10%) vs. male: *n =* 6 (3%), *p* = 0.002). Long-term survival (Figure 2) was also similar between male and female patients (log rank: *p* = 0.425).

Regression analysis. Age in years (OR: 1.026, *p* = 0.049), an acute pathology (OR: 1.941, *p* = 0.031) and preoperative dialyses (OR: 3.499, *p* = 0.010) were predictive for long-term mortality in our Cox regression model, sex (*p* = 0.466) was not. The full model is shown in Table 5.

## 4. Discussion

Our study’s most important findings can be summarised as: (i) Female patients are older but reveal fewer cardiovascular risk factors; they have aneurysms more often than male patients. (ii) However, as female patients undergo fewer concomitant cardiac procedures, their operative times are shorter. (iii) While survival and outcomes were similar between male and female patients, postoperative new in-stent thrombus formation was significantly more common in female patients.

Almost two-thirds of patients in our cohort undergoing aortic arch replacement were male. This is in line with other studies investigating aortic [9,10,11] and non-aortic cardiac surgery [3,4], all reporting a predominance of male patients. It is generally assumed that the female hormone estrogen has a protective effect on cardiovascular health but it lessens after menopause [12]. This is probably why females tend to develop cardiovascular disease at a later date and are therefore older than men, not only in our cohort, but in most studies addressing cardiac and aortic surgery [3,4,9,10,11]. We also observed that male patients had a significantly higher incidence of coronary artery disease and renal impairment, indicating a higher burden of arteriosclerosis.

Type A dissections were significantly more frequent in male patients. There is evidence of a preponderance of aortic dissections in younger male patients, both thoracic and thoracoabdominal [11,13]. Women seem to suffer from aortic dissections later in life. In fact, around 75% of patients with type A dissection under 40 years are male. The cut-off for a balanced gender distribution in type A dissection seems to be around 75 years [14]. This is in line with a recent paper investigating type A dissections in young adults under 30 years of age, presenting with 75% male patients [15]. In our cohort, male patients required emergency operation for acute type A or type B dissection significantly more often. Women suffering from acute aortic dissections often present with atypical symptoms, which leads to a delayed diagnosis [9,16] and may prevent timely arrival in the hospital to undergo surgical therapy. However, in our cohort, significantly more female patients presented with an aortic aneurysm involving the arch for elective FET implantation.

Men were more likely to require concomitant coronary artery bypass graft (CABG) during FET implantation, which reflects their increased arteriosclerosis burden. Male patients also underwent concomitant root replacement significantly more often, potentially because of the higher number of aortic dissections. Previous studies have shown that cardiopulmonary bypass time for FET procedures take longer in patients with aortic dissections, compared to aortic aneurysms [17,18]. Zheng et al. could even show that longer cardiopulmonary bypass time for FET procedures have a negative effect on 90-day mortality [19]. On the other hand, Beckmann et al. demonstrated that concomitant root replacement does not increase the perioperative risk in patients who undergo FET implantation for acute aortic dissection [19]. Selective antegrade cerebral perfusion times are equal between the two groups because the valved conduit or valve sparring aortic root replacements are implanted while the patients are cooled down.

Although many studies have reported a higher risk for women undergoing cardiac and aortic surgery [3,4,9,20], survival and outcomes were similar between genders in our cohort. We identified no sex- related difference in postoperative complications, in-hospital mortality or long-term survival. It seems like women’s higher age is offset by males’ higher incidence of cardiovascular risk factors and acute dissections. Another causative factor for similar survival despite sex differences may be that older patients tend to receive less extensive surgery with shorter duration of the procedure. Because the younger male population in our study presented with more acute dissections and received more complex surgery compared to the older women, the direct comparisons between sexes may be difficult. Therefore, there are differences between sexes with respect to the underlying disease and complexity of the necessary procedures. The fact that no differences in mortality were identified may be attributed to a type II error or heterogeneity between groups.

Postoperative new in-stent thrombus formation was significantly more common in female patients. This phenomenon was described earlier [21] and may be attributable to different aortic compliance and more turbulent flow in the female aorta. Other studies, examining this phenomenon of intraluminal thrombus formation after FET implantation, identified degenerative aneurysms, stent-graft diameter index, anticipated endoleak Ib, history of autoimmune disease and longer frozen elephant trunk stent-graft as potential risk factors. Therapeutic anticoagulation, however, seems to be protective [22,23]. The localization of the thrombus was, in the majority of cases, in the distal half of the FET stent-graft. We observed embolic complications with occlusion of the coeliac trunk, the superior mesenteric artery, the ileocolic artery and the renal arteries. All patients with thrombus formation received therapeutic anticoagulation and some patients were treated with an additional stent-graft to cover the thrombus. Until now, we did not change our anticoagulation concept after FET implantation because of the small number of events. Hence, there is an obvious need for in-depth analyses of perioperative coagulation management in larger case series. In summary, although male and female outcomes were similar in this study, there are sex-related differences in epidemiology and the clinical management of aortic diseases that require more investigation to be fully understood.

Limitations and strengths. Our study is limited by its small sample size, retrospective nature, confounding factors, risk of type II error, external validity (single high-volume center) and high heterogeneity of the cohort. However, this investigation contributes valuable knowledge on sex-specific differences in patients requiring FET total arch replacement.

## 5. Conclusions

Although female patients requiring FET total arch replacement are older, they present with fewer cardiovascular risk factors than male patients. Aneurysms are more common in female patients than male patients. However, as female patients require concomitant surgical procedures less often, their operative times are shorter. While our cohort’s survival and outcomes were similar, postoperative new in-stent thrombus formation was significantly more common in female patients.

## Figures and Tables

**Table 1:** Patient characteristics.

	All Patients (*n =* 362)	Female (*n =* 129)	Male (*n =* 233)	*p*
Age (years)	67 (59, 74)	68 (60, 77)	65 (58, 73)	0.012
Diabetes mellitus type 2	11 (3)	1 (1)	10 (4)	0.105
Hyperlipidemia	127 (35)	38 (29)	89 (38)	0.133
Hypertension	309 (85)	106 (82)	203 (87)	0.326
Coronary artery disease	111 (31)	28 (22)	83 (36)	0.008
History of smoking	159 (44)	50 (39)	109 (47)	0.133
COPD	34 (9)	17 (13)	17 (7)	0.088
History of stroke	41 (11)	16 (12)	25 (11)	0.604
Renal impairment	43 (12)	8 (6)	35 (15)	0.017
Bicuspid aortic valve	14 (4)	2 (2)	12 (5)	0.151
Connective tissue disease	29 (8)	11 (9)	18 (8)	0.840

Values are *n* (%) or median (first quartile, third quartile). COPD; chronic, obstructive pulmonary disease.

**Table 2:** Preoperative characteristics.

	All Patients (*n =* 362)	Female (*n =* 129)	Male (*n =* 233)	*p*
Redo case	151 (42)	46 (36)	105 (45)	0.118
Type A dissection *	139 (38)	40 (31)	99 (42)	0.042
Acute	64 (18)	14 (11)	50 (21)	0.014
Type B dissection	42 (12)	19 (15)	23 (10)	0.170
Acute	25 (7)	15 (12)	10 (4)	0.009
Type non-A-non-B dissection	44 (12)	17 (13)	27 (12)	0.737
Acute	29 (8)	19 (15)	10 (4)	1.000
Aneurysm	115 (32)	50 (39)	65 (28)	0.025
PAU	29 (8)	9 (7)	20 (9)	0.690
Other	8 (2)	1 (1)	7 (3)	0.269

Values are *n* (%) or median (first quartile, third quartile). PAU, penetrating aortic ulcer * including chronic residual dissections.

**Table 3:** Surgical details.

	All Patients (*n =* 362)	Female (*n =* 129)	Male (*n =* 233)	*p*
Cannulation				
Axillary	340 (94)	119 (92)	221 (94)	0.802
Valved conduit	43 (12)	9 (7)	34 (15)	0.041
VSARR	33 (9)	8 (6)	25 (11)	0.186
AVR	56 (15)	23 (18)	33 (14)	0.361
CABG	54 (15)	13 (10)	41 (18)	0.088
Beating heart	67 (19)	27 (21)	40 (17)	0.395
CPB time (min)	210 (176, 252)	189 (162, 232)	218 (184, 261)	<0.001
CX time (min)	120 (94, 158)	117 (87, 143)	125 (96, 168)	0.019
SACP time (min)	100 (70, 128)	99 (65, 125)	101 (81, 131)	0.603
Duration of surgery (min)	384 (328, 452)	361 (300, 417)	396 (345, 474)	<0.001
Lowest body temperature (°C)	25 (24, 25)	25 (24, 25)	25 (24, 25)	0.522
Perioperative transfusions				
PRBC	4 (2, 6)	4 (3, 6)	4 (2, 6)	0.058
FFP	5 (4, 8)	5 (4, 8)	6 (4, 8)	0.888
PC	4 (2, 4)	3 (2, 4)	4 (2, 4)	0.372

Values are *n* (%) or median (first quartile, third quartile). VSARR, valve sparring aortic root replacement; AVR, aortic valve replacement; CABG, coronary artery bypass graft; CPB, cardiopulmonary bypass; CX, aortic cross-clamp; SACP, selective antegrade cerebral perfusion; PRBC, packed red blood cell units; FFP, fresh frozen plasma units; PC, platelet concentrate units.

**Table 4:** Outcome characteristics.

	All Patients (*n =* 362)	Female (*n =* 129)	Male (*n =* 233)	*p*
Dialysis	39 (11)	13 (10)	26 (11)	0.861
Tracheotomy	23 (6)	6 (5)	17 (7)	0.380
Paraplegia	6 (2)	2 (2)	4 (2)	1.000
Stroke	56 (15)	20 (16)	36 (15)	1.000
Non-disabling	19 (5)	7 (5)	12 (5)	1.000
New in-stent thrombus *	19 (5)	13 (10)	6 (3)	0.002
In-hospital mortality	33 (9)	9 (7)	24 (10)	0.346

Values are *n* (%) or median (first quartile, third quartile). * in postoperative computed tomography angiography.

**Table 5:** Cox regression: mortality.

Variable	*p*	OR	95% CI
Male sex	0.466	1.247	0.688–2.260
Age (years)	0.049	1.026	1.000–1.052
Acute pathology	0.031	1.941	1.064–3.541
Redo case	0.067	1.724	0.963–3.087
Preoperative dialysis	0.010	3.499	1.356–9.025

OR, odds ratio; CI, confidence interval.

## Data Availability

The datasets discussed in this article are not readily available because of our institutional review board’s requirements. Reasonable individual requests will be evaluated by the corresponding author.

## Abbreviations

ACT	Activated clotting time
CABG	Coronary artery bypass graft.
CTA	Computed tomography angiography.
FET	Frozen elephant trunk.
mRS	modified Rankin Scale.
TEM	Type: Entry site, Malperfusion.
OR	odds ratio.
